# Dysregulation of secondary bile acid metabolism precedes islet autoimmunity and type 1 diabetes

**DOI:** 10.1016/j.xcrm.2022.100762

**Published:** 2022-10-03

**Authors:** Santosh Lamichhane, Partho Sen, Alex M. Dickens, Marina Amaral Alves, Taina Härkönen, Jarno Honkanen, Tommi Vatanen, Ramnik J. Xavier, Tuulia Hyötyläinen, Mikael Knip, Matej Orešič

**Affiliations:** 1 Turku Bioscience Centre, University of Turku and Åbo Akademi University, 20520 Turku, Finland; 2 School of Medical Sciences, Örebro University, 702 81 Örebro, Sweden; 3 Department of Chemistry, University of Turku, 20520 Turku, Finland; 4 Walter Mors Institute of Research on Natural Products, Federal University of Rio de Janeiro, 21941-599 Rio de Janeiro, Brazil; 5 Research Program for Clinical and Molecular Metabolism, Faculty of Medicine, University of Helsinki, Helsinki, Finland; 6 The Liggins Institute, University of Auckland, Auckland, New Zealand; 7 The Broad Institute of MIT and Harvard, Cambridge, MA, USA; 8 School of Science and Technology, Örebro University, Örebro, Sweden; 9 Pediatric Research Center, Children’s Hospital, University of Helsinki and Helsinki University Hospital, 00290 Helsinki, Finland

**Keywords:** bile acid, genome-scale metabolic modeling, gut microbiome, lipid metabolism, microbial metabolism, islet autoimmunity, type 1 diabetes, metabolomics, lipidomics

## Abstract

The gut microbiota is crucial in the regulation of bile acid (BA) metabolism. However, not much is known about the regulation of BAs during progression to type 1 diabetes (T1D). Here, we analyzed serum and stool BAs in longitudinal samples collected at 3, 6, 12, 18, 24, and 36 months of age from children who developed a single islet autoantibody (AAb) (P1Ab; n = 23) or multiple islet AAbs (P2Ab; n = 13) and controls (CTRs; n = 38) who remained AAb negative. We also analyzed the stool microbiome in a subgroup of these children. Factor analysis showed that age had the strongest impact on both BA and microbiome profiles. We found that at an early age, systemic BAs and microbial secondary BA pathways were altered in the P2Ab group compared with the P1Ab and CTR groups. Our findings thus suggest that dysregulated BA metabolism in early life may contribute to the risk and pathogenesis of T1D.

## Highlights

• The study defines systemic bile acid trajectories in early life
• Altered bile acid-metabolizing microbial pathways precede islet autoimmunity
• Secondary bile acid metabolism may contribute to the pathogenesis of type 1 diabetes

## Graphical abstract

**Figure:**
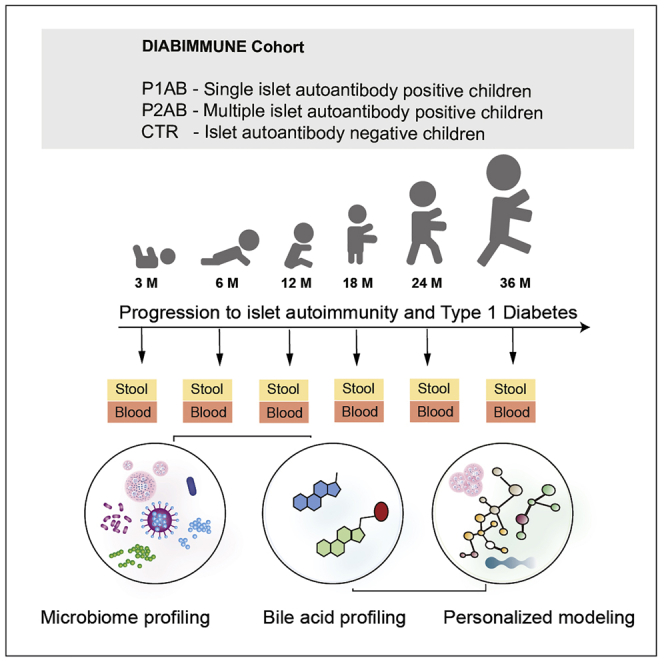

## Teaser

Lamichhane et al. combine shotgun metagenomics and metabolomics to show that systemic bile acids and microbial secondary bile acid pathways are altered early in children that develop islet autoantibodies. This suggests that dysregulation of bile acid metabolism in early life contributes to the risk and pathogenesis of type 1 diabetes.

## Introduction

Bile acids (BAs) are amphiphilic molecules that are crucial physiological agents for facilitating the absorption of lipids in the small intestine. BAs are produced from cholesterol in the liver. Primary BAs such as cholic acid (CA) and chenodeoxycholic acid (CDCA) are conjugated with either glycine or taurine in hepatocytes.^1^ Gut microbes transform primary BAs to secondary BAs in the intestine.^2^ Most of these BAs are re-absorbed back into the liver, while approximately 5% of the total BA pool is excreted via feces. Under normal physiological conditions, a small fraction (about 10%) of BAs are re-circulated and enter the systemic (enterohepatic) circulation, where they act as ligands for receptors in various peripheral tissues, including the farnesoid X receptor (FXR) and the membrane receptor known as Takeda G protein-coupled membrane receptor (TGR5).^3^, ^4^, ^5^ FXR and TGR5 signaling plays a critical role in regulation of systemic lipid, glucose, and energy homeostasis.^6^^,^^7^ Dysregulation of systemic BA metabolism has been linked to multiple diseases, including fatty liver disease, cardiovascular disease, and type 2 diabetes.^6^^,^^8^^,^^9^ Thus, the gut microbiome-BA axis is increasingly recognized as a therapeutic target for treating metabolic and immune-mediated disorders.^9^, ^10^, ^11^

Previous metabolomics and gut microbiome studies suggest that children who progress to islet autoimmunity and type 1 diabetes (T1D) later in life are characterized by disturbances in lipid metabolism^12^, ^13^, ^14^, ^15^ and gut microbiota,^16^, ^17^, ^18^ suggesting that there is an interplay among host metabolism, the immune system,^19^ and the gut microbiome during early T1D pathogenesis. However, our current understanding of both microbial and host regulatory BA pathways in the development of islet autoimmunity remains limited.

Herein, we set out to investigate how microbial BA pathways are regulated in children who develop islet autoimmunity. We analyzed BAs and subject-matched microbiome profiles in a prospective series of samples, which included children who developed multiple autoantibodies (P2Ab) during follow-up and are thus at high risk for progression to T1D later in life,^20^ and those children who developed only one islet autoantibody (P1Ab) but did not progress to T1D during follow-up. We also included control children (CTRs) who remained islet autoantibody (AAb) negative during follow-up.

## Results

### Prospective study of bile acids and gut microbiome in children at risk for T1D

**Figure 1:**
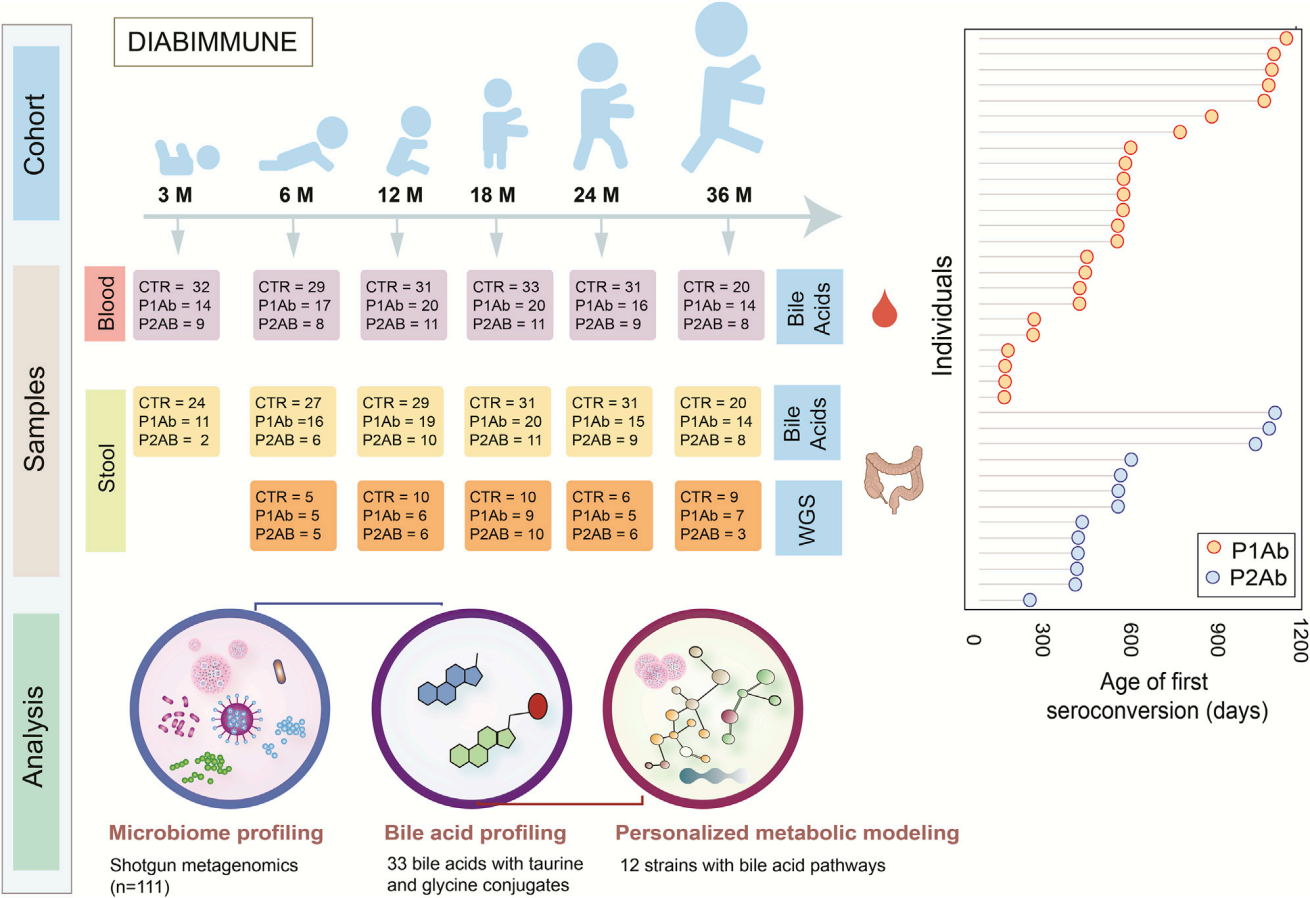
Outlines of analytical study flow This illustrates the number of serum and stool samples collected for targeted BA measurement and matched stool samples for metagenomics analysis (whole-genome sequencing [WGS]) at each time point. Here, we analyzed BAs in a longitudinal series of serum and stool samples collected at 3, 6, 12, 18, 24, and 36 months of age from children who developed a single islet autoantibody (P1Ab; n = 23) or multiple islet autoantibodies (P2Ab; n = 13) and controls (CTRs; n = 38) who remained autoantibody (AAb) negative during follow-up. The samples were stratified into P1Ab, P2Ab, and CTR groups. Moreover, the figure shows the age of seroconversion among the children taking part in this study.

In a longitudinal study setting, we analyzed BAs in subject-matched stool (n = 304) and serum (n = 333) samples from three study groups: P1Ab (n = 23), P2Ab (n = 13), and CTR (n = 38) (Figure 1). From each child, we analyzed stool and serum samples at six different time points corresponding to the ages of 3, 6, 12, 18, 24, and 36 months. A total of 33 BAs, including both primary (glycine/taurine conjugates) and secondary BAs were assayed (STAR Methods). Previously published stool shotgun metagenomics data (whole-genome shotgun sequencing [WGS]) from a subset of children (n = 111 stool samples in total)^18^ were included in the study (Figure 1). Table S1 includes the demographic characteristics of the study population.

### Age-related changes in bile acid and microbiome profiles

**Figure 2:**
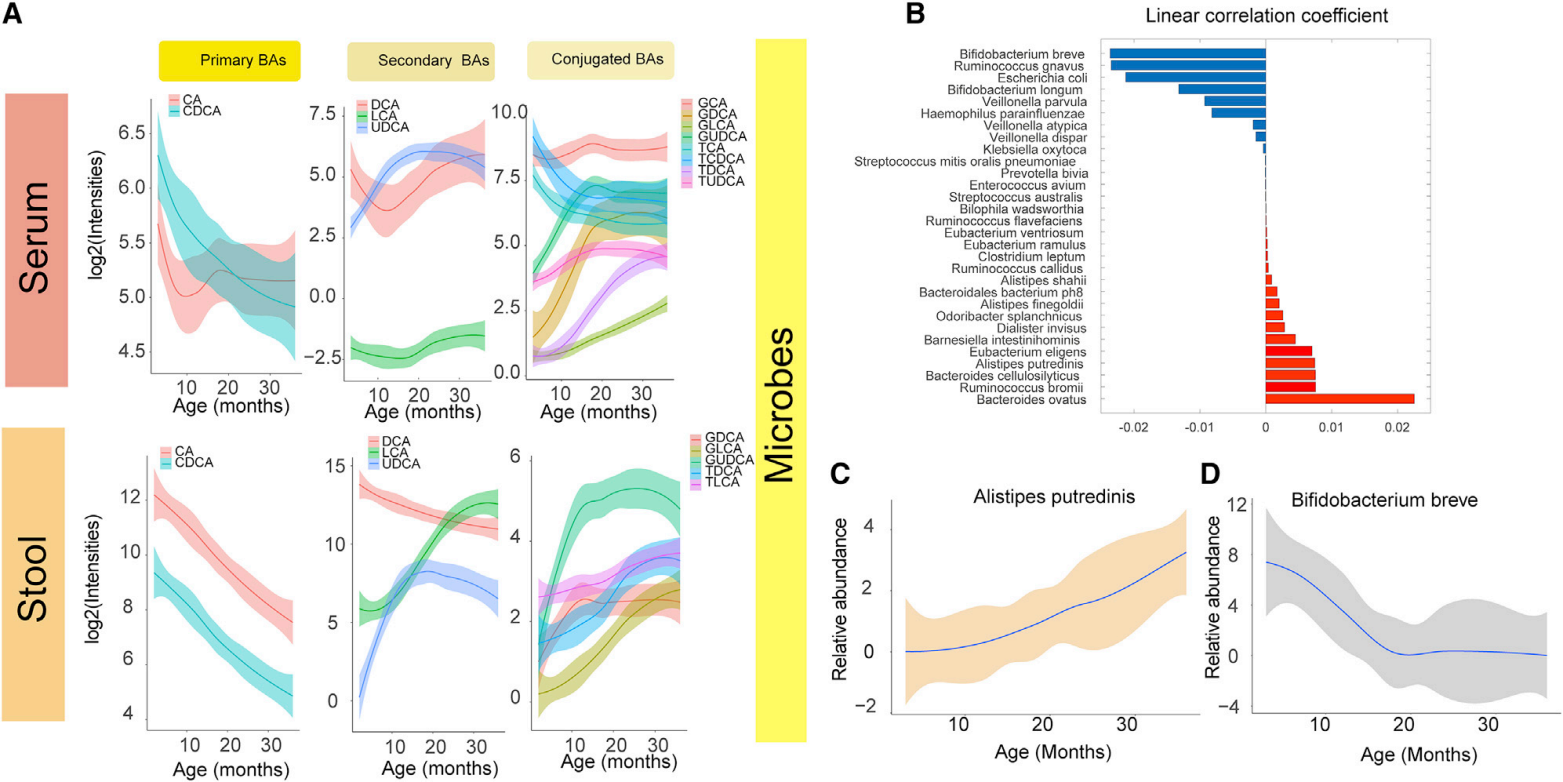
Age-related changes in bile acids and microbiome (A) The loess curve plot of BAs over time (3, 6, 12, 18, and 24) between stool and serum samples. This panel plots, separately, representative primary, secondary, and conjugated BAs that changed significantly over time (p < 0.05). (B) Bar plots showing correlation coefficients for the association between age and different microbes. Red represents inverse correlations, while blue represents positive correlations as obtained by multivariate linear regression using the R package MaAsLin2. (C and D) The loess curve plot of selected microbes over time. Here, in a longitudinal study setting, we analyzed BAs in subject-matched stool (n = 304) and serum (n = 333) samples from three study groups: P1Ab (n = 23), P2Ab (n = 13), and CTR (n = 38). Whole-genome shotgun sequencing was available from a subset of children (n = 111 stool samples in total).

In order to determine the contributions of various factors to subjects’ BA profiles, multivariate associations were tested for by applying linear models using covariates of age, gender, and case status (P1Ab, P2Ab, or CTR), taking into account random effects within an individual sample/subject (STAR Methods). Age showed the strongest impact on BA profile (23 stool and 21 serum BAs at p < 0.05; Figure 2A), while five BAs in stool and one serum BA were different across case groups, and one stool and four serum BAs were different between the genders (Tables S2 and S3). Primary BAs, including cholic acid and chenodeoxycholic acid, were decreased both in stool and in circulation with increasing age (Figure 2A). A similar trend was seen for deoxycholic acid (DCA), a secondary BA. Low levels of other secondary BAs (including their taurine and glycine conjugates) were observed during early infancy (3 and 6 months), which steadily increased at/after the first year of life (12 and 18 months) and remained stable at 24 and 36 months of age (Figure 2A).

Gut microbial profiles followed the dynamic BA trajectories (Figures 2A and 2B; Table S4). Multivariate associations were tested for by applying linear models using covariates including age, gender, case (P1Ab, P2Ab, or CTR), exclusive breastfeeding status, and age at introduction of solid food, taking into account random effects within an individual sample or subject. Age was the strongest factor associated with the composition of the infant gut microbiome (Figure S1). Several microbial species, at the strain level, were associated (n = 30, p < 0.05) with age (Figure 2B; Table S4); dominated by *Ruminococcus*, *Alistipes*, and *Eubacterium* species, which showed an increasing trend with age (Figure 2C). However, this did not stabilize at 36 months of age. On the other hand, the abundances of six of 17 microbes, including *Bifidobacterium breve* (Figure 2D), remained lower during 3–12 months of infancy and stabilized at 24 and 36 months of age.

### Alteration of the gut microbiome and bile acid metabolism associates with progression to islet autoimmunity

**Figure 3:**
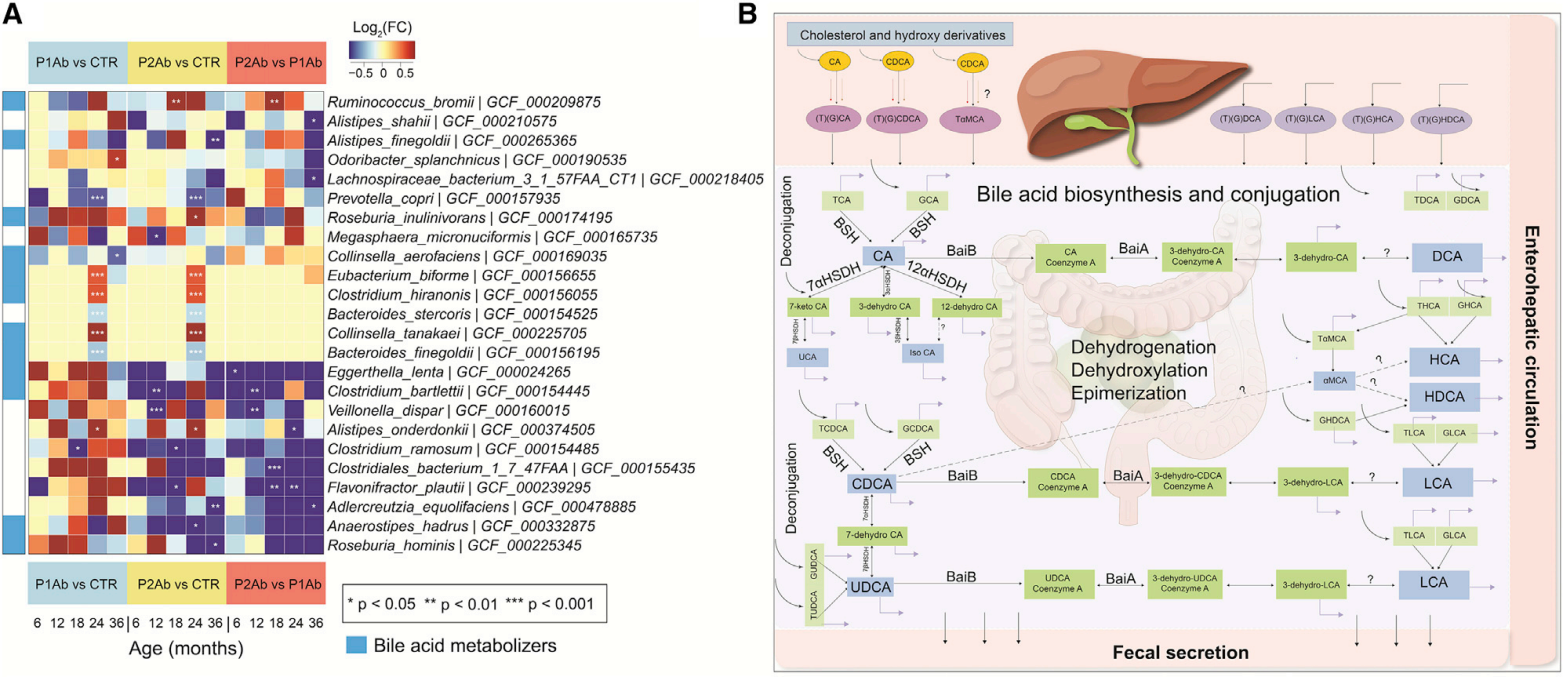
Microbial strains in progression to islet autoimmunity (A) A heatmap showing the log_2_ fold changes (FCs) in the strain-level abundances of the gut microbes in P1Ab versus CTR, P2Ab versus CTR, and P2Ab versus P1Ab groups at 6, 12, 18, 24, and 36 months of the follow-up (n = 111). Red, blue, and yellow denote increase, decrease, and no change in the abundances between the differential conditions, respectively. Statistical significance was assessed using ANCOVA adjusted for “diet variables” as covariates and p value adjusted for FDR < 0.05. Microbes with BA pathways (annotated by the AGORA compendium) are marked with light blue color. (B) An illustration of BA metabolism and related pathways in humans. Whole-genome shotgun sequencing was available from a subset of children (n = 111 stool samples in total). Question marks indicate “unknown enzymes.” Single- and double-headed arrows represent irreversible and reversible reactions, respectively.

Differential analysis showed that 24 microbial strains were altered (analysis of covariance [ANCOVA]; adjusted p values for false discover rate [FDR] < 0.05) among the study groups (P1Ab and/or P2Ab and/or CTRs) at least at one time point (STAR Methods; Figure 3A). Of note, 12 of 24 microbial strains were known to exhibit BA metabolic pathways as annotated by the Assembly of Gut Organisms through Reconstruction and Analysis (AGORA) compendium.^21^, ^22^, ^23^ Among these, *Alistipes*, *Clostridium*, *Eggerthella*, *Ruminococcus*, *and Roseburia* strains were altered between the P1Ab and P2Ab groups (Figure 3A). Lower abundances of *Clostridium and Eggerthella* strains and increased abundances of *Ruminococcus* strains were apparent in P2Ab (versus P1Ab/CTR) group at 18 months and/or 24 months of age. The BA pathways exhibited by these microbes include ten different reaction classes that can carry out deconjugation, dehydrogenation, dehydroxylation and epimerization of BAs in the human gut (Figures 3B and S2).

### Regulation of secondary BA pathways before the emergence of islet autoantibodies

In order to understand the interplay between the gut microbiome and BA biotransformation in the progression to islet autoimmunity, we developed personalized community microbiota models for each child (STAR Methods). The community microbiota model comprises 12 abundant microbial strains and their BA reactions (Figures 3A and S2).

**Figure 4:**
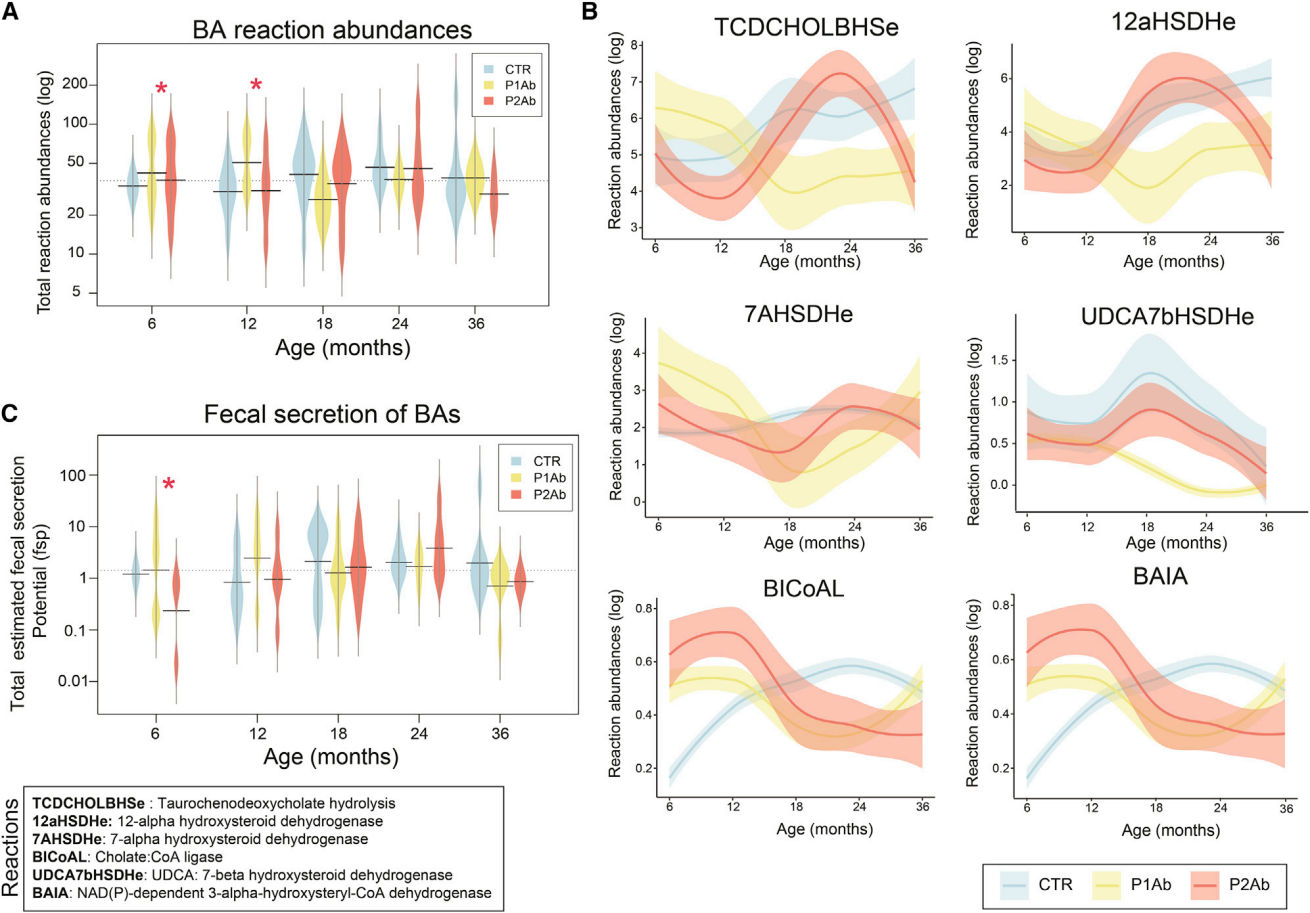
Regulation of bile acid reactions in progression to islet autoimmunity (A–C) Bean plots showing the levels of total BA reaction abundances and the total fecal secretion potentials (FSPs) predicted by the community microbiota models in the CTR, P1Ab, and P2Ab groups at 6, 12, 18, 24, and 36 months of follow-up. The black dotted line denotes the mean of the population. The black dashes in the bean plots represent the group mean. Asterisk denotes significant differences (ANOVA with Tukey’s HSD, adjusted p < 0.05). (B) Locally weighted scatterplot smoothing (LOWESS) plot showing the longitudinal trend of an individual BA reaction abundance in the CTR (light blue), P1Ab (yellow), and P2Ab (orange) groups during follow-up. The shaded area around the curves depicts the 95% confidence interval. Whole-genome shotgun sequencing was available from a subset of children (n = 111 stool samples in total).

The community microbiota modeling suggested that the total BA reaction abundances were markedly decreased (ANOVA, Tukey’s honestly significant difference [Tukey’s HSD], adjusted p < 0.05) in the P2Ab versus P1Ab group at 6 and 12 months of age, i.e., before the median age of seroconversion (Figure 4A). Moreover, at this age, the predicted abundances of bile salt hydrolases (*BSH*) reaction(s) decreased in the P2Ab versus P1Ab group. However, the abundances of these reactions peaked at 24 months of age (post-seroconversion) (Figure 4B). At this age, several reactions in the alpha/beta dehydroxylation pathway, particularly cholate ligases (*BICoAL*, *BAIA*), showed decreased abundances in the P2Ab group (versus P1Ab). 7-Alpha/beta hydroxylation pathways aid in the production of secondary BAs (e.g., DCA, HCA, HDCA, LCA) from primary BAs (e.g., CA, CDCA), respectively (Figures 3B and 4B).

The community modeling also suggested that the total fecal secretion potential (FSP) of secondary BAs was significantly decreased (Tukey’s HSD, adjusted p < 0.05) in the P2Ab (versus P1Ab) group at 6 and 12 months of age (Figure 4C). Taken together, secondary BA production appears to be decreased in the P2Ab group compared with the P1Ab and/or CTR group(s) with the emergence of islet autoantibodies.

### Targeted measurements of BAs revealed a decrease in secondary BA levels in progression to islet autoimmunity

**Figure 5:**
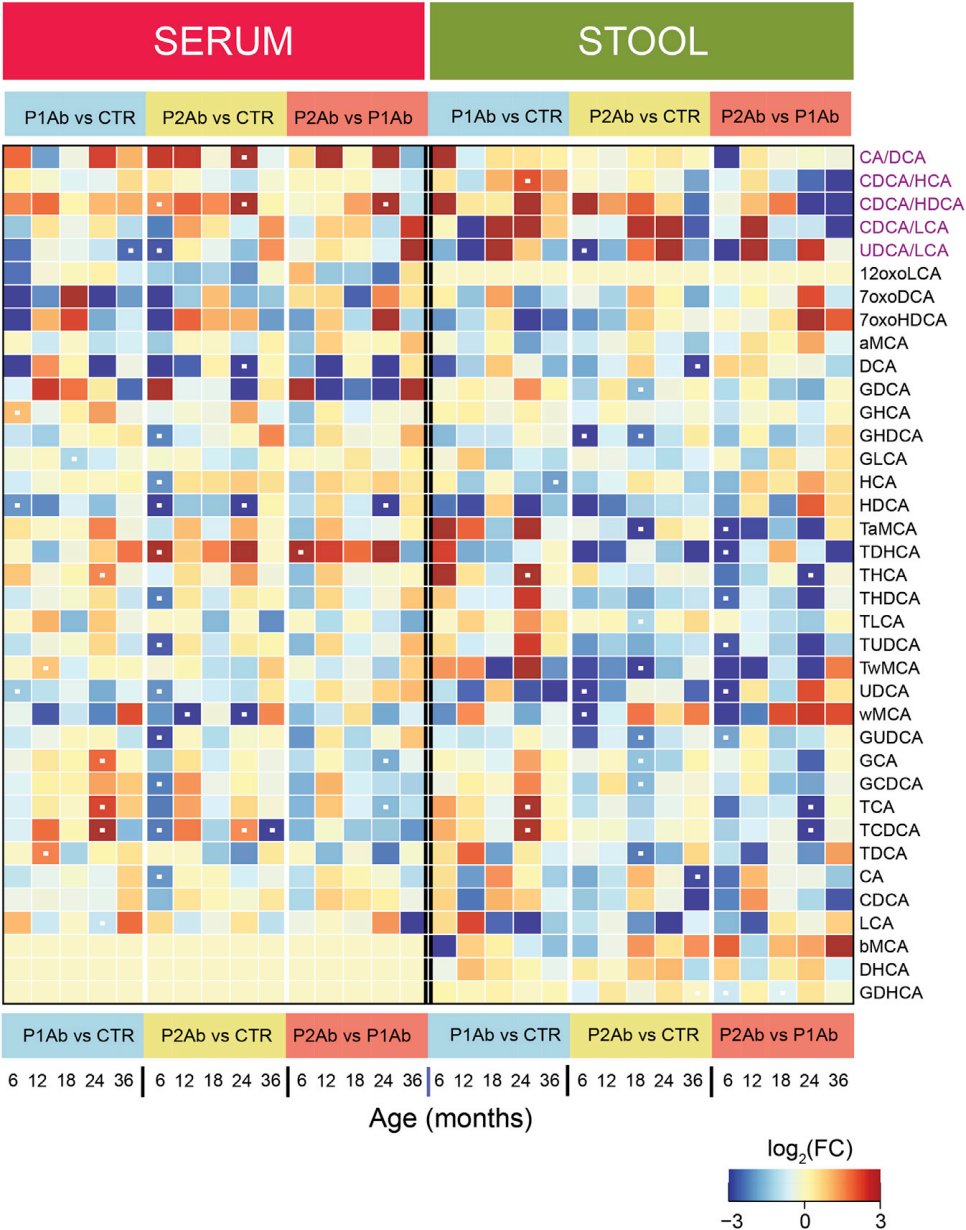
Systemic alterations in bile acid profiles in progression to islet autoimmunity Heatmap showing the log_2_ fold changes (FCs) in BA profiles in P1Ab versus CTR, P2Ab versus CTR, and P2Ab versus P1Ab groups at 6, 12, 18, 24, and 36 months of follow-up. Red, blue, and yellow denote increase, decrease, and no change in the intensities of BAs between the differential conditions, respectively. Statistical significance was estimated using ANCOVA adjusted for “diet variables” as covariates, p adjusted for FDR < 0.05.

We sought to determine specific BA concentration differences between the study groups (P1Ab and/or P2Ab and/or CTRs) in the longitudinal series of stool and serum samples. We observed differences in the concentrations of BAs among the study groups. In particular, taurine and glycine conjugates of secondary BAs (e.g., THDCA, TUDCA, UDCA, GUDCA, GDHCA) were decreased in the P2Ab (versus P1Ab or CTR) group at 6 months of age in the stool samples (Figure 5). At this age, UDCA and its conjugates were also altered between P2Ab and CTR groups in the serum samples. Intriguingly, the ratio of UDCA/LCA was decreased in the P2Ab versus CTR group in stool and serum samples (Figure 5). Changes in the primary BAs (CA and CDCA) and their conjugates were apparent at 24 months of age, i.e., post-seroconversion in the stool samples (Figures 5 and 3B). Of note, differences in the concentrations of BAs were less pronounced in the serum samples compared with the stool samples (Figure 5). Correlation between the BAs in both stool and serum samples of the CTR, P1Ab, and P2Ab groups are shown in Figure S3.

### Association between BAs levels in the stool and gut microbial strains showed association in the P2Ab group

**Figure 6:**
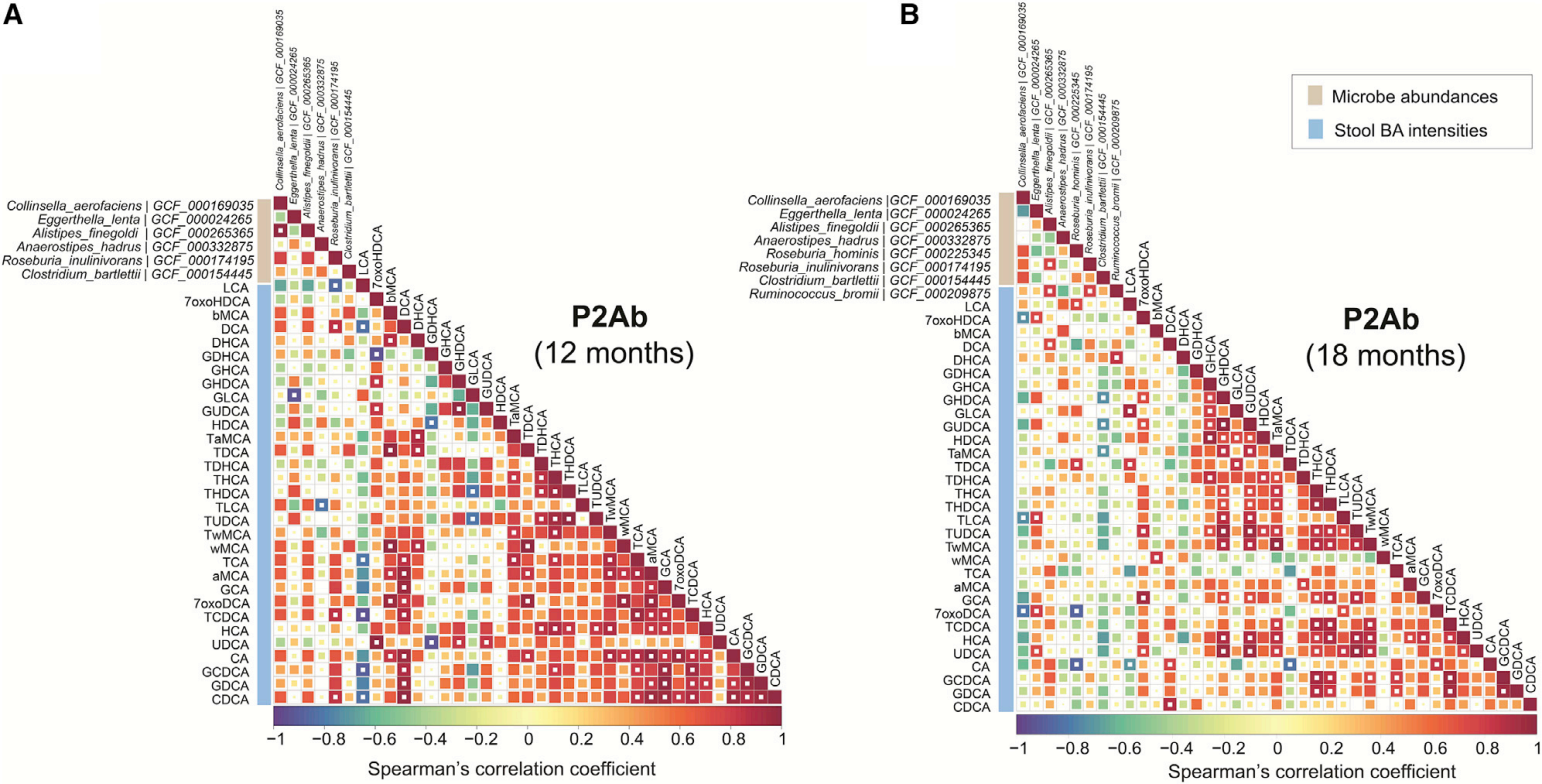
Cross-correlation between the gut microbiome and systemic (stool) levels of BA in progression to islet autoimmunity Correlation plots showing bivariate Spearman’s correlations between the gut microbiome (metagenomics) and level of BAs (lipidomics) in the stool samples of the P2Ab (n = 111) group at (A) 12 and (B) 18 months. Red, blue, and white/yellow represent positive, negative, and no correlation, respectively. The white “dot” indicates that the correlation is statistically significant (p adjusted for FDR < 0.05). Here, in a longitudinal study setting, we analyzed BAs in subject-matched stool (n = 304) and serum (n = 333) samples from three study groups: P1Ab (n = 23), P2Ab (n = 13), and CTR (n = 38). Whole-genome shotgun sequencing was available from a subset of children (n = 111 stool samples in total).

Next, we studied whether stool BA profiles associated with the microbiome in the longitudinal series. *Eggerthella lenta* was positively correlated with UDCA and its conjugates at 12 and/or 18 months of age. Intriguingly, at these time points, *Eggerthella lenta* was negatively correlated with GLCA and positively correlated (adjusted p values for FDR < 0.05) with TLCA, respectively. *Clostridium bartlettii* showed negative associations with (GUDCA, GHDCA, TaMCA) and GDHCA at 18 and 24 months of age, respectively (Figures 6B and S4). *Anaerostipes hadrus* and *Roseburia hominis* strains were associated with LCA and its conjugate at 12 and/or 18 months of age (Figures 6A and 6B).

## Discussion

By combining targeted metabolomics and metagenomics data, we were able to demonstrate that host-microbial BA co-metabolism is dysregulated in the progression to islet autoimmunity and overt T1D. Our findings suggest that children who progress to multiple islet autoantibodies (P2Ab) during follow-up, and are thus at high risk for developing T1D later in life,^20^ have distinct and persistently altered systemic BA concentrations and species abundances in the gut microbiome, compared with those children who develop, at most, a single islet autoantibody or those who remained negative for islet antibodies (CTR) during follow-up.

Our results reveal that children who progressed to multiple islet autoantibodies later in life had decreased concentrations of conjugated BAs in early life. In line with this, TUDCA, a conjugated secondary BA, was observed to reduce the incidence of diabetes development by improving the glucose utilization and metabolism in streptozotocin-treated C57BL/6 mice.^24^ Our personalized community modeling of the gut microbiota identified specific differences in the BA pathways of P2Ab versus P1Ab or CTR groups. Several intermediary reactions of 7-alpha/beta hydroxylation and bile salt hydrolase pathways were altered at or before the age of seroconversion. Alteration of relative abundances of BSH levels are associated with the occurrence and development of various diseases in humans.^25^ Here, we revealed that the activity of BSH may be related to the development of islet autoimmunity and risk for clinical T1D. Furthermore, our results show that in the P2Ab (versus P1Ab) group, reaction abundances of 7-alpha/beta hydroxylation pathway remained lower at 6 and 12 months of age, which gradually increased at later time points. BSH pathways are key gatekeepers of BA transformation in the gut.^26^ We found that the stool concentrations of secondary BAs, particularly UDCA, DCA, HDCA, and their glycine and/or taurine conjugates, were downregulated in P2Ab versus P1Ab and/or CTR groups. Furthermore, decreased fecal secretion potentials of the secondary BAs in the P2Ab group during early life further support the view that a decrease in secondary BA levels at or before the age of seroconversion might occur because of a decrease in the metabolic potential of the microbiota-encoded 7-alpha/beta hydroxylation pathway, which aids in the transformation of secondary BAs.

BA metabolism is a cooperative process between host and the microbiome.^2^ We found the prevalent gut microbe *Eggerthella lenta* was associated with the stool concentrations of TLCA and GLCA in the P2Ab study group. *Eggerthella lenta* hydroxysteroid dehydrogenases is capable of using both tauro- and glyco-conjugated BAs as substrates.^27^ Intriguingly, *Eggerthella lenta* has previously been associated with human disease and found to activate T-helper type 17 (Th_17_) immune cells.^28^^,^^29^ Our results also suggest *Eggerthella lenta* as an immunomodulatory microbe; however, the mechanisms of how this specific microbe contribute to autoimmunity progression remains still to be elucidated.

We also found that BA concentrations were strongly associated with the age of the children. In agreement with previous findings,^30^^,^^31^ we observed that the abundances of gut microbes (with the exception of a few strains) gradually increased with the age of the children. Interestingly, many of these microbes are involved in the biotransformation of BAs.^21^^,^^22^ Recently, a study characterized the age-dependent gut microbial and metabolic changes in the murine gastrointestinal tract,^32^ where BAs were identified as a major driver for the early maturation of the gut microbiome.

Future immunological and metabolic studies are needed to elucidate how disturbances in the gut microbiome-BA axis contribute to the initiation of T1D. It is known that administration of secondary BAs, specifically LCA, a secondary BA, causes downregulation of circulating lipids^33^, similar to previous findings in progression to T1D.^12^^,^^13^^,^^15^ Altered secondary BA metabolism may also disrupt immune homeostasis.^34^ Recently, two distinct derivatives of LCA, including 3-oxoLCA and isoallo LCA, were found to affect host immune responses by directly modulating T cell differentiation, potentially via gut microbial activity.^35^ Given the increasingly recognized immunomodulatory role of secondary BAs, it is plausible that future studies aimed at elucidation of the three-way interaction among gut microbes, BAs, and the host immune system, may pave the way to new strategies for disease prevention.

In summary, our findings suggest that dysregulated BA metabolism in early life may contribute to the risk and pathogenesis of T1D. BA metabolism may also be an underlying link between the gut microbiome and host (lipid) metabolism during the period preceding seroconversion to positivity for islet autoantibodies and overt T1D.

### Limitations of the study

We acknowledge some limitations of our study, such as the small sample size. Nevertheless, we report BA changes in a longitudinal setting, defining the time course of changes in BA metabolism including with respect to the onset of islet autoimmunity. However, it is clear that these findings need to be replicated in larger studies and in heterogeneous populations. We also acknowledge that the present analysis is exploratory and therefore requires further validation and mechanistic studies. In particular, the functional role of the BA metabolizers as identified in this study should be tested by *in vitro* and/or *in vivo* experiments. Furthermore, future studies will need to quantify the expression of BSH enzyme in stool samples.

## STAR★Methods

### Key resources table

**Table:** 

REAGENT or RESOURCE	SOURCE	IDENTIFIER
**Biological sample**		

Serum	This paper	N/A
Fecal	This paper	N/A
**NIST 1950**	Sigma-Aldrich	NIST® SRM® 1950

**Chemicals, peptides, and recombinant proteins**		

**Cholic acid (CA)**	Sigma-Aldrich	Cat#700212P; CAS: 81-25-4
**Chenodeoxycholic acid (CDCA)**	Sigma-Aldrich	Cat#C9377; CAS: 474-25-9
**Deoxycholic acid (DCA)**	Sigma-Aldrich	Cat#700197P; CAS: 302-95-4
**Dehydrocholic acid (DHCA)**	Sigma-Aldrich	Cat#30830; CAS: 81-23-2
**Glycocholic acid (GCA)**	Sigma-Aldrich	Cat#G2878; CAS: 1192657-83-2
**Glycochenodeoxycholic acid (GCDCA)**	Sigma-Aldrich	Cat#G0759; CAS: 16564-43-5
**Lithocholic acid (LCA)**	Sigma-Aldrich	Cat#L6250; CAS: 434-13-9
**Taurocholic acid (TCA)**	Sigma-Aldrich	Cat#T4009; CAS: 345909-26-4
**Taurochenodeoxycholic acid (TCDCA)**	Sigma-Aldrich	Cat#T6260; CAS: 6009-98-9
**Taurodeoxycholic acid (TDCA)**	Sigma-Aldrich	Cat#T0557; CAS: 207737-97-1
**Taurodehydrocholic acid (TDHCA)**	Sigma-Aldrich	Cat#700242P; CAS: 517-37-3
**(Taurohyodeoxycholic acid) THDCA**	Sigma-Aldrich	Cat#T0682; CAS: 38411-85-7
**Taurolithocholic acid (TLCA)**	Sigma-Aldrich	Cat#T7515; CAS: 6042-32-6
**(Tauroursodeoxycholate) TUDCA**	Sigma-Aldrich	Cat#T0266; CAS: 35807-85-3
**Hyocholic acid (HCA)**	Sigma-Aldrich	Cat#700159P; CAS: 547-75-1
**α-Muricholic acid (αMCA)**	Sigma-Aldrich	Cat#700232P; CAS: 2393-58-0
**β-Muricholic Acid (βMCA)**	Sigma-Aldrich	Cat#SML2372; CAS: 2393-59-1
**ω-Muricholic Acid (ωMCA)**	Sigma-Aldrich	Cat#700231P; CAS: 6830-03-1
**12-oxo-lithocholic acid (12-oxo-LCA)**	Sigma-Aldrich	Cat#SMB00913; CAS: 5130-29-0
**Tauro-β-muricholic acid (TβMCA)**	Sigma-Aldrich	Cat#700244P; CAS: 25696-60-0
**Tauro-ω-Muricholic acid (TωMCA)**	Sigma-Aldrich	Cat#700245P; CS-0119476
**Tauro-α-muricholic acid (TαMCA)**	Steraloids	Cat#C1893-000; CAS: 25613-05-2
**7-oxodeoxycholic acid (7-oxo-DCA)**	Steraloids	Cat#C1250-000; CAS: 911-40-0
**Hyodeoxycholic Acid (HDCA)**	Steraloids	Cat#C0860-000; CAS: 83-49-8
**Taurohyocholic Acid (THCA)**	Steraloids	Cat#C1887-000; CAS: 32747-07-2
**Glycodehydrocholic acid (GDHCA)**	Steraloids	Cat#C2020-000; CAS: 3415-45-0
**Glycohyocholic Acid (GHCA)**	Steraloids	Cat#C1860-000; CAS: 32747-08-3
**Glycohyodeoxycholic acid (GHDCA)**	Steraloids	Cat#C0867-000; CAS: 13042-33-6
**Glycolithocholic acid (GLCA)**	Steraloids	Cat#C1435-000; CAS: 474-74-8
**Glycoursodeoxycholic acid (GUDCA)**	Steraloids	Cat#C1025-000; CAS: 64480-66-6
**Glycodeoxycholic acid (GDCA)**	Steraloids	Cat#C1085-000; CAS: 16409-34-0
**Ursodeoxycholic acid (UDCA)**	Steraloids	Cat#C1020-000; CAS: 128-13-2
**Cholic-2,2,4,4-d4 Acid (CA-d4)**	Qmx Laboratories	Cat#D-2452; CAS: 116380-66-6
**Lithocholic-2,2,4,4-d4 Acid (LCA-d4)**	Qmx Laboratories	Cat#D-3742; CAS: 83701-16-0
**Ursodeoxycholic Acid D4 (UDCA-d4)**	Qmx Laboratories	Cat#BX5231; CAS: 347841-46-7
**Chenodeoxycholic-2,2,4,4-d4 Acid (CDCA-d4)**	Qmx Laboratories	Cat#D-2772; CAS: 99102-69-9
**Deoxycholic-2,2,4,4-d4 Acid (DCA-d4)**	Qmx Laboratories	Cat#D-2941; CAS: 112076-61-6
**Glycocholic acid-2,2,4,4-d4 (GCA-d4)**	Qmx Laboratories	Cat#ACA-160819-0032; CAS: 1201918-15-1
**Glycolithocholic-2,2,4,4-d4 Acid (GLCA-d4)**	Qmx Laboratories	Cat#D-6318; CAS: 2044276-16-4
**Glycoursodeoxycholic-2,2,4,4-d4 Acid (GUDCA-d4)**	Qmx Laboratories	Cat#D-6319; CAS: 2044276-17-5
**Glycochenodeoxycholic-2,2,4,4-d4 acid (GCDCA-d4)**	Qmx Laboratories	Cat#ACA-160819-0033; CAS: 1201918-16-2
**Taurocholic acid -d4**	Synthesized in our laboratory	^36^

**Deposited data**		

**Raw and analyzed data**	This paper	Submitted to the Metabolomics Workbench repository

**Software and algorithms**		

**MultiQuant 3.0.3**	Sciex	https://sciex.com/products/software/multiquant-software
**Analyst 1.6.3**	Sciex	https://sciex.com/products/software/analyst-software
**R (v4.0.4)**	packages used: Scater, RcmdrMisc, MaAsLin2, Heatmap.2, boxplot, beanplot, gplot, ggplot2	https://www.r-project.org/
**MATLAB (v R2017a)**	COBRA, Microbiome Modeling Toolbox, Recon3D	https://www.mathworks.com/ (Heirendt et al., 2017, Brunk et al., 2018)

### Resource availability

#### Lead contact

Further information and requests for resources and reagents should be directed to and will be fulfilled by the lead contact, Matej Orešič (matej.oresic@oru.se).

#### Materials availability

This study did not generate new unique reagents.

### Experimental model and subject details

#### Human subjects

The DIABIMMUNE study recruited 832 families in Finland (Espoo), Estonia (Tartu), and Russia (Petrozavodsk) with infants carrying HLA alleles which confer risk for autoimmunity. The subjects involved in the current study were chosen from the subset (n = 74) of available samples (matched serum and stool) in the international DIABIMMUNE study children who progressed to at least a single AAb (P1Ab, n = 23), who progressed to multiple islet AAb (P2Ab, n = 13), and controls (CTRs, n = 38), i.e. the children who remained islet AAb-negative during the follow-up in a longitudinal series of samples collected at 3, 6, 12, 18, 24 and 36 months from each child. Here no prior sample-size estimation was performed. The study groups were matched for HLA-associated diabetes risk, gender, country and period of birth. This study was conducted according to the guidelines in the Declaration of Helsinki. The Ethics and Research Committee of the participating Universities and Hospitals approved the study protocol. All families provided written informed consent prior to sample collection. Table S1 includes the anthropometric characteristics of the study population.

### Method details

#### Quantification of bile acids

The BAs were measured in serum and fecal sample as described previously.^37^^,^^38^ All fecal sample were freeze-dried prior to extraction to account for the inconsistency in the fecal water content and dry weight in the stool. Briefly, 20 μL of serum, or fecal homogenate (prepared by adding 1:20 (m/v) ultrapure water to 50 mg of feces) was filtered through a Ostro Protein Precipitation and Phospholipid Removal 96-well plate (Waters Corporation, Milford, USA), using 100 μL of cold methanol contemning the internal standard mixtures (LCA-d4, TCA-d4, GUDCA-d4, GCA-d4, CA-d4, UDCA-d4, GCDCA-d4, CDCA-d4, DCA-d4, GLCA-d4). The eluent was collected and evaporated to dryness and the residue was re-suspended in 20 μL of a 40:60 MeOH: H2O v/v mixture. The analyses were performed on an ACQUITY HSS T3 (2.1 × 100 mm, 1.8 μm) column, Waters (Milford), coupled to a triple quadrupole mass spectrometer (Waters Corporation, Milford, USA) with an atmospheric electrospray interface operating in negative ion mode. Separation was performed using gradient elution with 0.1% formic acid in water (v/v) (A) and 0.1% formic acid in acetonitrile:methanol (3:1, v/v) (B) at a flow rate of 0.5 mL/min. Gradient program was 0 min 15% B, 0–1 min; 30% B, 1–16 min; 16–18 min; 70% B, 18–23 min 100% B, and equilibrium time between runs was 7 min. The injection volume was 5 μL and the column was kept at 35°C. An external calibration with nine calibration points (0.0025–600 ng/mL) was carried out for use in quantitation.

For quality control, we randomized the order of samples and injected pooled 1) quality control (QC) 2) a blank sample and 3) a known standard every 10 samples. In addition to that the samples were blinded to the person preparing and running the experiments.

#### Analysis of islet autoantibodies

Four diabetes-associated autoantibodies were analyzed from each serum sample with specific radiobinding assays: insulin autoantibodies (IAA), glutamic acid decarboxylase antibodies (GADA), islet antigen-2 antibodies (IA-2A), and zinc transporter 8 antibodies (ZnT8A) as described previously.^39^ Islet cell antibodies (ICA) were analyzed with immunofluoresence in those subjects who tested positive for at least one of the autoantibodies. The cut-off values were based on the 99th percentile in non-diabetic children and were 2.80 relative units (RUs) for IAA, 5.36 RU for GADA, 0.78 RU for IA-2A and 0.61 RU for ZnT8A.

### Quantification and statistical analysis

#### Sequencing and phylogenetic profiling of human gut microbiota

Metagenomic shotgun sequencing was conducted as previously described.^18^ Raw metagenomic sequencing data was retrieved from (https://diabimmune.broadinstitute.org/) (NCBI BioProject ID: PRJNA231909).^18^ Stool samples (n = 111) were common between the published metagenomics data^18^ and the stool BAs measured in the present study. Metagenomic data from the matched samples (n = 111) were considered for further analysis.

As stated in^18^, host genome‒contaminated reads and low-quality reads are already removed from the raw sequencing data using kneadData v0.4. Taxonomic microbiome profiles were determined using MetaPhlAn2^40^ using default parameters.

#### Genome-scale community modeling of human gut microbiota

Previously, genome-scale metabolic modeling (GSMM) using an Assembly of Gut Organisms through Reconstruction and Analysis (AGORA) approach has been used to elucidate the role of gut microbiota in BA biotransformation in humans.^22^ In addition, it has been used to estimate the metabolic capabilities of gut microbes and related pathways under different biological conditions.^21^

We used GSMM to model the dynamics of BA metabolism aided by human gut microbiota under various conditions. In order to reduce the complexity of community modeling, we included genome-scale metabolic models (GEMs) of 12 abundant gut microbial strains that have BA metabolic pathways, and were significantly (ANCOVA; p.adjusted for FDR <0.05) altered between the study groups (P1Ab, P2Ab and CTRs), at least at one time-point (Figure 3A). All the microbial-GEMs obtained were retrieved from the *‘AGORA_BA’* compendium (v1.03)^21^^,^^22^ stored at the Virtual Metabolic Human Database (VMH)^23^ and assessed for further analysis.

Next, we developed personalized community models for each individual by contextualizing the community microbiota model with the metagenomic abundances of the microbes estimated for each individual/sample. The microbial strains (GEMs) were coupled into a community microbiota model. A detailed protocol for integration of metagenomic abundances into a community microbiota model has been described elsewhere.^21^^,^^22^^,^^41^

Metabolic reconstruction such as Recon3D,^42^ the small intestinal epithelial cells (sIECs)^43^ model, and the VMH database and bibliographic references were mined, and putative BA transporters in the human gut were identified. The BA transporters and exchange reactions were added. Recon3D as a host model was coupled with the community microbiota model using the *‘createMultipleSpeciesModel’* function coded in MMT, subsequently the flux coupling constraints were added. A compartment ‘[b]’ for body fluids was introduced. Sanity checks were performed using the COBRA Toolbox.

All the personalized microbiota models developed were able to carry out basic metabolic tasks, including exchange and transport of BAs. The average reactions and metabolites of a microbiota community model was 15,800 and 13,900 respectively. These models were simulated and results were divided for three different study groups (P1Ab, P2Ab and CTRs). GSMM was performed using the COBRA Toolbox^44^ and the Microbiome Modeling Toolbox (MMT)^41^ deployed in MATLAB *Inc*., version R2017a.

The fecal secretion potential (FSP) of a BA reaction is given by

(Equation 1) $$FSPij=A_{i}\times v_{j}$$

where ‘FSP_ij_’ denotes the estimated potential of ‘j^th^’ BA in ‘i^th^’ species. A and v represent the relative abundance of a species and absolute flux potential (mmol/gDw/day), respectively.^22^^,^^45^ FSP determines the metabolic efficiency of a particular reaction, under a specified condition. The total FSP determines the metabolic capability/potential of the gut microbes in a community to perform a particular task. Likewise, BA reaction abundances in a community model was estimated by the *‘calculateReactionAbundance’* function coded in MMT.^22^^,^^23^

#### Statistical analysis

The R statistical programming language (v4.0.4) and MATLAB *Inc*., (vR2017a) was used for data analysis. The *‘Heatmap*.*2’*, *‘boxplot’*, *'beanplot'*, ‘*gplot*’, and ‘*ggplot2*’ R libraries/packages were used for data visualization.

#### Impact of clinical/demographic factors on stool microbiome

The effect of different factors such as age, gender, presence of antibodies, age of T1D onset, duration of breast feeding, HLA-risk class on the microbiome abundances were evaluated for each sample, and the % of explained variance (EV) was estimated. The data were log_2_-transformed, centered to zero mean and unit variance (auto scaled). The relative contribution of each factor to the total variance in the dataset was estimated by fitting a linear regression model, where the normalized abundances of the microbes were regressed to the factor of interest, and thereby median marginal coefficients (R^2^) were estimated. This analysis was performed using the *‘Scater’* package in R (v4.0.4). Age was found to be a confounding factor (>10% EV).

#### Differential abundance analysis of the microbiome and BAs

The metagenomic and BA data were log_2_-transformed. By combining analysis of covariance (ANCOVA) adjusted for ‘diet’ variables (total length of breast feeding, length of exclusive breast feeding and time of introduction of solid foods) used as covariates, and statistical significance was determined by multiple testing adjusted for FDR at p < 0.05. We were able to identify differentially abundant microbes (adjusted p < 0.05) between a paired conditions (*e*.*g*. P2Ab *vs*. CTRs). This analysis was performed by *‘aov’* functions deployed in the *‘stats’* package (R v4.0.4). Multivariable associations using linear models were performed using ‘MaAsLin2′ R package.^46^ The locally-weighted regression plot was made using smoothing interpolation function loess available from ggplot2 package in R. Loess regression was performed using *‘loess’* function deployed in the *‘stats’* package (R v4.0.4).

#### Bivariate correlation analysis

*RcmdrMisc’* package was used to estimate Spearman’s correlation between the BA intensities in the stool, community BA exchange reaction potentials, and related microbial abundances. The p values were adjusted for FDR at (adjusted p < 0.05). Results are plotted using *‘heatmap*.*2’* function of *‘gplots’* package (v.3.0.4).

## Data Availability

• Metagenomic sequencing data can be downloaded from https://diabimmune.broadinstitute.org/diabimmune/ (NCBI BioProject ID: PRJNA231909). • The targeted bile acid metabolomics datasets generated in this study is available at the NIH Common Fund’s National Metabolomics Data Repository (NMDR) website, the Metabolomics Workbench (https://www.metabolomicsworkbench.org) where it has been assigned Study ID ST001992 and ST001991. The data can be accessed directly at Metabolomics Workbench: https://doi.org/10.21228/M86D99. This work is supported by NIH grant U2C-DK119886. • Scripts and codes for GSMM can be downloaded from: https://github.com/parthosen/Diab_GSMM_T1D. • Any additional information required to reanalyze the data reported in this work paper is available from the lead contact upon request.

## Supplemental information

Document S1. Figures S1–S4 and Tables S1–S4

Document S2. Article plus supplemental information
