# Supplementary material for: Dysregulation of secondary bile acid metabolism precedes islet autoimmunity and type 1 diabetes
Source: Cell Rep Med. 2022 Oct 3;3(10):100762. doi: 10.1016/j.xcrm.2022.100762 (PMC9589006; doi:10.1016/j.xcrm.2022.100762)
Supplement: Document S1. Figures S1–S4 and Tables S1–S4 [file mmc1.pdf]

Cell Reports Medicine, Volume *3*

Supplemental information

Dysregulation of secondary bile acid metabolism

precedes islet autoimmunity and type 1 diabetes

Santosh Lamichhane, Partho Sen, Alex M. Dickens, Marina Amaral Alves, Taina Härkönen, Jarno Honkanen, Tommi Vatanen, Ramnik J. Xavier, Tuulia Hyötyläinen, Mikael Knip, and Matej Orešič

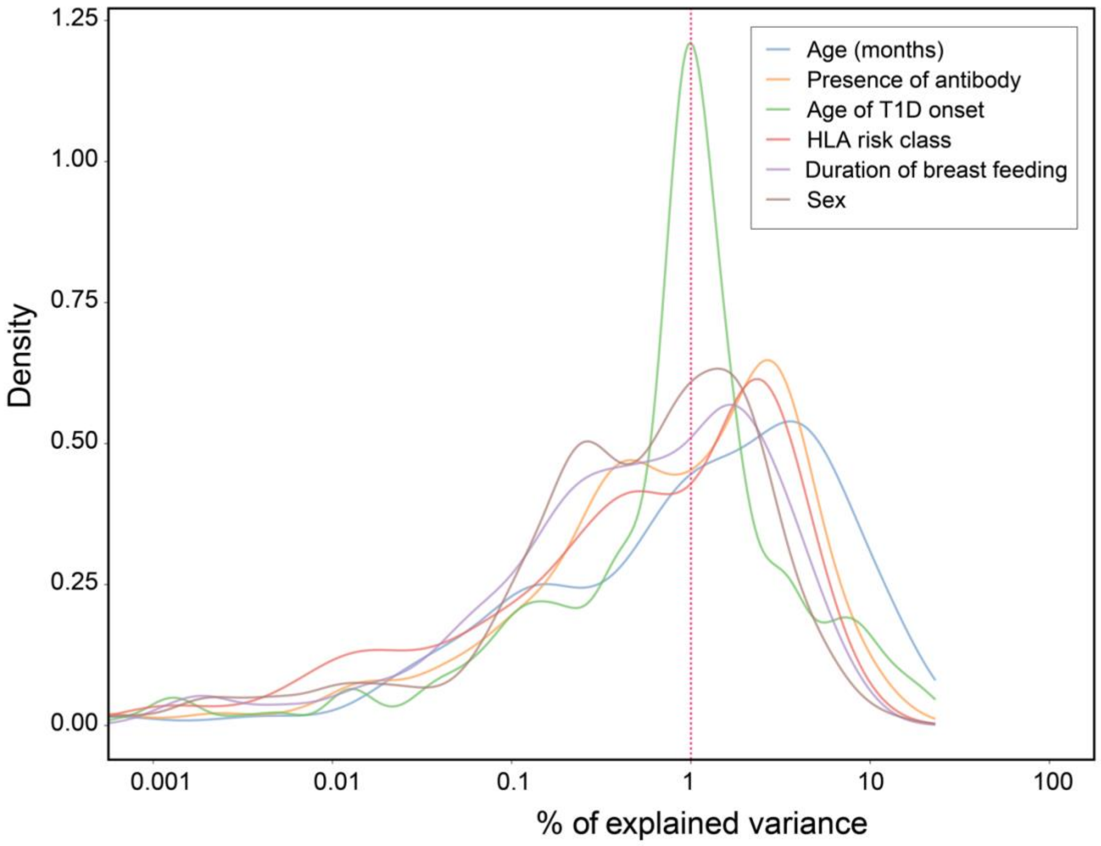

Figure S1. Factor analysis and identification of confounding factors affecting the microbiome. A density plot showing sample-wide distribution of (% of explained variances (EVs)) of various clinical and demographic factors associated with the normalized metagenomic analyzed in (n=111) stool samples. Related to **Figure 3**.

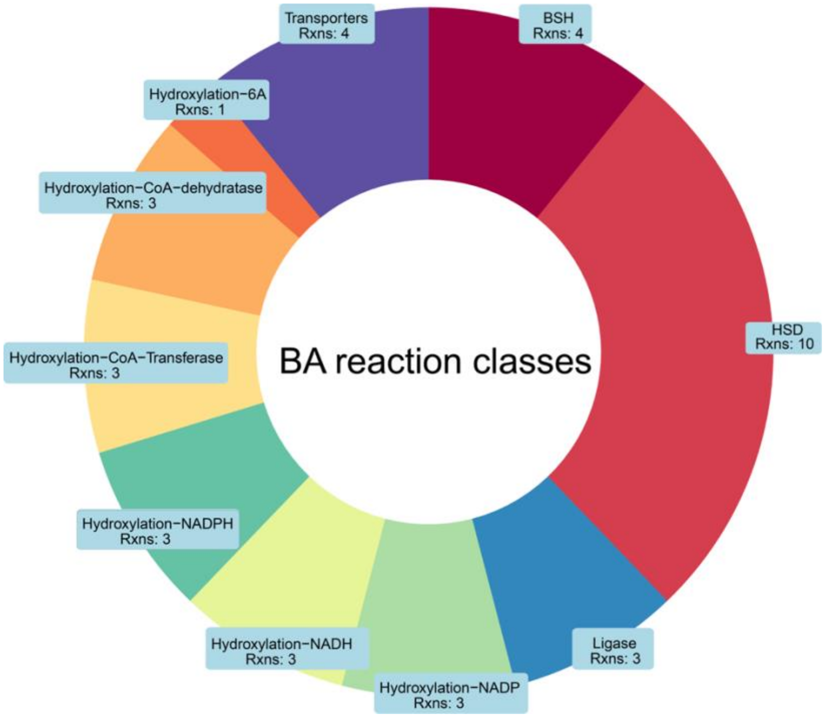

Figure S2. Bile acid reaction classes exhibited by the 25 abundant microbes. The BA reactions/pathways exhibited by human gut microbes spans between 10 different reaction classes that can carry out deconjugation, dehydrogenation, dehydroxylation and epimerization of BAs in the gut. Related to **Figures 3** and **4**.

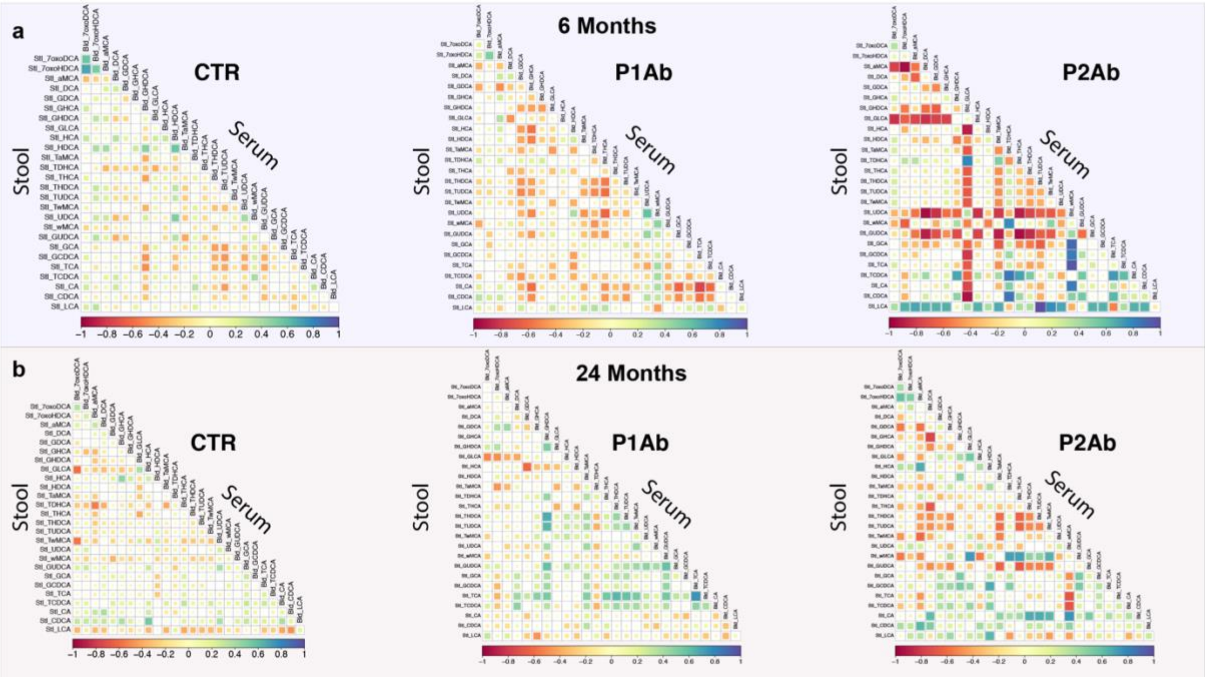

Figure S3. Correlation between the BAs in the stool vs. plasma samples. Correlation plots showing bivariate Spearman's correlations between the BAs in the stool and plasma samples of CTR, P1Ab and P2Ab groups at, **a**) 6 months and **b**) 12 months. Red, blue and white/yellow color represents positive, negative and no correlation respectively. Color bar denotes strength of Spearman's correlations. Related to **Figure 5** and **6.**

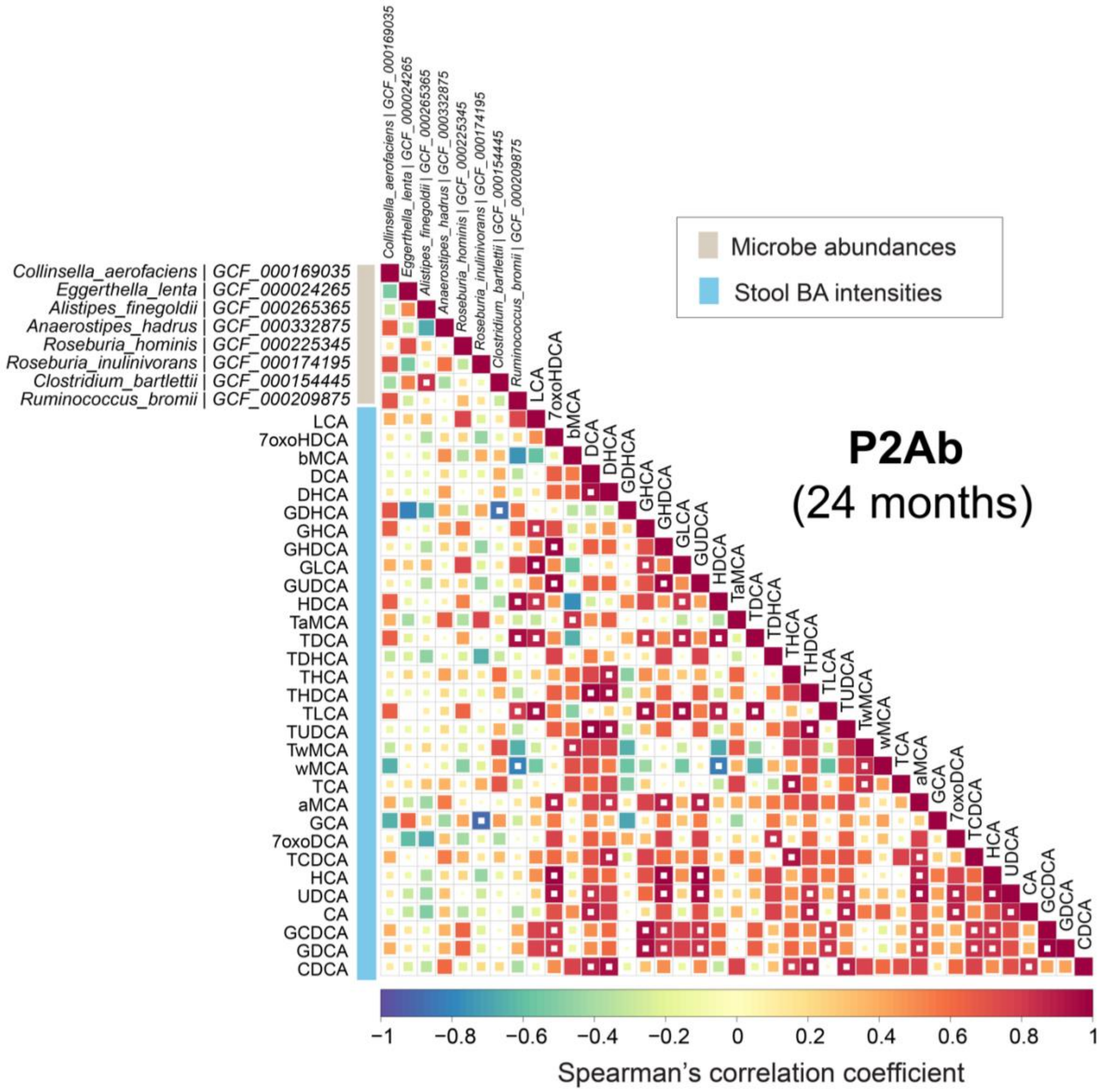

Figure S4. Cross-correlation between the gut microbiome and BA systemic (stool) levels in progression to islet autoimmunity. Correlation plots showing bivariate Spearman's correlations between the gut microbiome (metagenomics) and level of BA (lipidomics) in the stool samples of P2Ab group at 24 months. Red, blue and white/yellow color represents positive, negative and no correlation respectively. The white 'dot' depicts that the correlation is statistically significant (p.adjusted < 0.05). Related to **Figure 6.**

Table S1. Demographic characteristics of study population. Related to **Figure 1**.

	P1Ab	P2Ab	CTR
Gender (girls, boys)	(17, 7)	(8, 5)	(25, 13)
Country (Finland, Estonia)	(14, 10)	(10, 3)	(23, 15)
Breast Feeding length exclusive (days) (mean ± SD)#	(64.09 ± 67.93)	(85.90 ± 72.31)	(73.88 ± 70.72)
Age of Seroconversion (days) (mean ± SD)	(600.66 ± 348.83)	(561.80 ± 297.67)	NA

included for available subgroup of subjects, respectively: P1Ab = 21, P2Ab = 10, CTR = 34.

Table S2. Multivariate associations using linear models were performed with co-variates including age, sex, and case status (P2Ab or P1Ab or CTRs) in stool. Related to **Figure 2**.

Metadata	Features	coefficient	P value	Q value
Age	LCA	1.765868	3.19E-35	3.95E-33
Age	HCA	-0.71767	1.96E-23	1.21E-21
Age	CA	-1.01563	2.19E-20	9.04E-19
Age	CDCA	-0.9442	3.85E-19	1.01E-17
Age	HDCA	1.017782	4.07E-19	1.01E-17
Age	GLCA	0.488118	5.37E-19	1.11E-17
Age	GDHCA	-0.18543	1.16E-15	2.05E-14
Age	TDCA	0.451116	1.21E-10	1.88E-09
Age	OXODCA	-0.58323	2.75E-10	3.79E-09
Age	UDCA	0.807496	1.29E-09	1.52E-08
Age	GUDCA	0.549565	1.35E-09	1.52E-08
Age	DCA	-0.5483	7.77E-09	8.03E-08
Age	THCA	-0.50224	8.87E-09	8.46E-08
Age	TaMCA	-0.64066	1.05E-08	9.29E-08
Age	GHDCA	0.531877	4.10E-08	3.39E-07
Age	bMCA	-0.5654	6.81E-08	5.28E-07
Age	TLCA	0.238253	5.38E-07	3.93E-06
Age	oxoHDCA	-0.42934	4.05E-05	0.000279
Age	GHCA	-0.1721	8.74E-05	0.00057
Age	TDHCA	-0.16553	0.000214	0.001328
Age	GCDCA	0.203357	0.001128	0.006658
Age	GDCA	0.203259	0.001189	0.006701
Age	aMCA	-0.1961	0.001382	0.007451
Case	GUDCA	-0.66431	0.009749	0.050371
Case	GHDCA	-0.6725	0.014903	0.073919
Case	TUDCA	-0.7258	0.026109	0.11991
Case	HDCA	-0.54519	0.028887	0.12793
Case	THDCA	-0.65175	0.044398	0.189841
sex	HDCA	-0.26414	0.017872	0.085236

Table S3. Multivariate associations using linear models were performed with co-variates including age, sex, and case status (P2Ab or P1Ab or CTRs) in serum. Related to **Figure 2**.

Metadata	Feature	Coefficient	P value	Q value
Age	TDCA	0.810951	4.84E-34	5.61E-32
Age	HCA	-0.615	5.16E-29	2.99E-27
Age	GLCA	0.366212	3.35E-25	1.29E-23
Age	TaMCA	-0.66722	7.47E-21	2.17E-19
Age	GUDCA	0.692191	2.46E-18	5.72E-17
Age	THCA	-0.4358	5.13E-16	9.92E-15
Age	UDCA	0.584086	6.06E-15	1.00E-13
Age	GHDCA	0.650438	2.03E-13	2.95E-12
Age	X7oxoHDCA	-0.33823	3.78E-11	4.88E-10
Age	GDCA	0.992672	7.50E-11	8.70E-10
Age	TLCA	0.16878	9.46E-10	9.98E-09
Age	HDCA	0.503645	4.96E-09	4.79E-08
Age	X7OXODCA	-0.35812	6.31E-08	5.63E-07
Age	TCA	-0.38511	1.41E-07	1.17E-06
Age	TwMCA	0.239079	1.54E-07	1.19E-06
Age	TCDCA	-0.54483	3.05E-07	2.21E-06
Age	CDCA	-0.28592	8.93E-07	6.10E-06
Age	GHCA	-0.24791	1.23E-06	7.92E-06
Age	TUDCA	0.24461	1.94E-06	1.19E-05
Age	THDCA	0.235196	2.78E-06	1.61E-05
Age	wMCA	-0.06713	0.007594	0.040039
Case	HDCA	-0.5611	0.02448	0.118321
sex	TDHCA	0.019688	0.004911	0.027125
sex	GHCA	0.159941	0.013417	0.067668
sex	GCA	0.16621	0.034349	0.159378
sex	THCA	0.137237	0.04182	0.186583

Table S4. Multivariate associations using linear models were performed with co-variates including age, sex, case (P1Ab, P2Ab, or CTR), exclusive breastfeeding status and age at introduction of solid food in the stool microbiome dataset. Related to **Figure 2**.

Metadata	Feature	Coefficient	P value	Q value
Age	t__GCF_000296465	0.004486	0.000367	0.188503
Age	t__GCF_000154465	0.007448	0.000423	0.188503
Age	t__Bifidobacterium_breve_unclassified	-0.02361	0.000437	0.188503
Age	t__Ruminococcus_gnavus_unclassified	-0.02345	0.000532	0.188503
Age	t__GCF_000311925	0.001729	0.001062	0.23215
Age	t__Veillonella_parvula_unclassified	-0.00926	0.001138	0.23215
Age	t__Escherichia_coli_unclassified	-0.02125	0.001593	0.257914
Age	t__GCF_000146185	0.007015	0.001626	0.257914
Age	t__Bacteroides_ovatus_unclassified	0.022542	0.001814	0.258973
Age	t__GCF_000190535	0.002589	0.00227	0.270116
Age	t__Streptococcus_mitis_oralis_ pneumoniae_unclassified	-9.42E-05	0.002459	0.270116
Age	t__GCF_000469345	0.00022	0.005303	0.359319
Age	t__GCF_000468015	0.000393	0.008339	0.410631
Age	t__GCF_000265365	0.002044	0.010483	0.45361
Age	t__Haemophilus_parainfluenzae_unclassified	-0.00818	0.013668	0.546632
Age	t__Bacteroides_cellulosilyticus_unclassified	0.007534	0.018335	0.638609
Age	t__GCF_000209875	0.007567	0.021438	0.704108
Age	t__GCF_000153885	0.000151	0.021695	0.704108
Age	t__Bifidobacterium_longum_unclassified	-0.0132	0.02991	0.827056
Age	t__GCF_000160055	0.002842	0.030971	0.827056
Age	t__GCF_000160015	-0.00151	0.032419	0.827056
Age	t__GCF_000185705	2.68E-05	0.036414	0.827056
Age	t__GCF_000154345	0.000272	0.037218	0.827056
Age	t__Klebsiella_oxytoca_unclassified	-0.00039	0.038761	0.827056
Age	t__Enterococcus_avium_unclassified	-6.51E-05	0.039959	0.827056
Age	t__Veillonella_atypica_unclassified	-0.00192	0.04004	0.827056
Age	t__Prevotella_bivia_unclassified	-8.01E-05	0.044712	0.827056
Age	t__GCF_000210575	0.000905	0.044771	0.827056
Age	t__Streptococcus_australis_unclassified	-5.03E-05	0.045701	0.827056
Age	t__Ruminococcus_flavefaciens_unclassified	5.76E-05	0.046551	0.827056
bfexclusive	t__GCF_000218445	-0.00023	0.011324	0.475598
bfexclusive	t__GCF_000173435	0.008964	0.035727	0.827056
Case	t__Bacteroides_ovatus_unclassified	0.074748	0.00066	0.188503
Case	t__GCF_000157995	0.000422	0.006098	0.359319
Case	t__GCF_000159975	0.008664	0.007215	0.380819
Case	t__GCF_000190535	-0.00842	0.016237	0.612361
Case	t__GCF_000239295	0.001214	0.018828	0.640137

Case	t__GCF_000205025	-0.00101	0.027401	0.827056
Case	t__GCF_000218385	0.000273	0.028564	0.827056
Case	t__GCF_000190535	-0.00645	0.030116	0.827056
Case	t__GCF_000169035	0.007233	0.033878	0.827056
Case	t__GCF_000242155	0.000466	0.033902	0.827056
Case	t__GCF_000020225	0.024661	0.036764	0.827056
sex	t__Streptococcus_mitis_oralis_pneumoniae_unclassified	-7.28E-05	0.017403	0.621277
sex	t__Parabacteroides_merdae_unclassified	0.008036	0.037341	0.827056
sex	t__Clostridium_symbiosum_unclassified	0.000931	0.040375	0.827056
sex	t__GCF_000157995	0.000114	0.048621	0.827056
solidfood	t__GCF_000156655	-7.68E-05	0.002109	0.270116
solidfood	t__GCF_000154845	0.00228	0.004443	0.359319
solidfood	t__GCF_000374585	0.000533	0.005726	0.359319
solidfood	t__Finegoldia_magna_unclassified	3.19E-05	0.006471	0.359319
solidfood	t__Anaerococcus_hydrogenalis_unclassified	1.62E-05	0.006471	0.359319
solidfood	t__GCF_000311745	0.000145	0.006471	0.359319
solidfood	t__Anaerococcus_prevotii_unclassified	1.60E-05	0.006471	0.359319
solidfood	t__Clostridium_sporogenes_unclassified	6.95E-05	0.006471	0.359319
solidfood	t__Lactobacillus_salivarius_unclassified	0.000288	0.006471	0.359319
solidfood	t__GCF_000163295	0.000388	0.006471	0.359319
solidfood	t__Lactobacillus_plantarum_unclassified	6.51E-05	0.006471	0.359319
solidfood	t__Lactococcus_garvieae_unclassified	0.003168	0.006542	0.359319
solidfood	t__Lactobacillus_oris_unclassified	0.000104	0.007467	0.380819
solidfood	t__Bacteroides_massiliensis_unclassified	0.005645	0.00946	0.442277
solidfood	t__Bacteroides_ovatus_unclassified	0.018634	0.009601	0.442277
solidfood	t__Lactobacillus_gasseri_unclassified	0.002302	0.010061	0.44898
solidfood	t__Lactobacillus_rhamnosus_unclassified	0.00106	0.013781	0.546632
solidfood	t__Leuconostoc_lactis_unclassified	4.80E-05	0.016295	0.612361
solidfood	t__Streptococcus_thermophilus_unclassified	0.00656	0.017259	0.621277
solidfood	t__Ruminococcus_lactaris_unclassified	0.002304	0.027957	0.827056
solidfood	t__GCF_000332875	0.000562	0.030928	0.827056
solidfood	t__Enterococcus_faecalis_unclassified	0.000492	0.035194	0.827056
solidfood	t__Bifidobacterium_longum_unclassified	-0.01271	0.03673	0.827056
solidfood	t__GCF_000157935	0.013253	0.039907	0.827056
solidfood	t__Prevotella_bivia_unclassified	-8.04E-05	0.046854	0.827056
